# Tracheotomy

**Published:** 1828-05

**Authors:** 


### TRACHEOTOMY.

A girl, nine years of age, whilst laughing, suffered a bean of a large size to pass into the trachea, which excited at intervals a convulsive cough, which continually increased in violence. She was brought to the Hospital at Berlin thirty-six hours after the accident. Fits of suffocation alternated with attacks of exhaustion, and there was a severe pain beneath the upper part of the sternum, increased at each inspiration. Although it was feared that the bean had reached the bronchi, an incision, one inch in length, was made in the lower part of the trachea: the speech was immediately suppressed, and a quantity of bloody mucus escaped from the wound. This relieved the patient: nevertheless, the bean could not be discovered either by a probe or straight forceps introduced into the bronchi; trials which could not be repeated, because each time the mucous membrane was touched produced a violent attack of suffocation, attended with universal spasms. The patient was bled, had a dose of opium given to her, and a piece of gauze was placed over the wound. She had a tolerable night; but the next day the attacks of suffocation returned with such violence that her life appeared in the greatest danger. The incision was therefore prolonged nearly to the upper edge of the sternum, and again an attempt was made to pass a small pair of forceps into the bronchi, but it caused a recurrence of the spasms and a convulsive cough, during which the epidermis of the bean became visible, but which disappeared instantly, before it could be laid hold of. It again showed itself, however, and was extracted: the length was nine lines by four and a half wide. All the bad symptoms disappeared: the wound healed in about a month; the speech was restored, and nothing remained but a hoarseness.—*Medical Gazette.*

